# Nutritional parameters and lifestyle practices of people who use drugs undergoing treatment for recovery in Lebanon: a descriptive study

**DOI:** 10.1017/jns.2021.9

**Published:** 2021-03-08

**Authors:** Nadine Mahboub, Rana Rizk, Nanne de Vries

**Affiliations:** 1 Department of Nutrition and Food Sciences, Lebanese International University, Beirut, Lebanon; 2 Department of Health Promotion, Maastricht University, Maastricht, The Netherlands; 3 Institut National de Santé Publique, d'Epidémiologie Clinique, et de Toxicologie (INSPECT-LB), Beirut, Lebanon

**Keywords:** Drug use disorder, Drug abuse treatment centres, Health promotion, Healthy lifestyle, Nutritional status, Lebanon

## Abstract

Drug use disorder is a major public health problem. Once people who use drugs (PWUD) are referred to treatment, addressing their lifestyle practices and improving their quality of life improves treatment outcomes. The present study assessed the nutritional status and lifestyle practices among PWUD undergoing treatment for recovery in Lebanon. Furthermore, it explored significant differences in these parameters depending on the offered treatment modality, namely opioid substitution treatment (OST) and rehabilitation. In total, 187 PWUD undergoing treatment for recovery participated in this cross-sectional study. Nutritional status and anthropometrics, dietary intake, nutrition knowledge, food addiction, biochemical parameters, sleep and physical activity were measured using validated tools. Of the participants, 88⋅8 % were well nourished based on the Subjective Global Assessment. In total, 67 % gained weight during treatment placing them in the overweight category. This increase in weight was significantly higher in the rehabilitation group. It came in parallel with higher protein and energy intakes, higher rate of food addiction, and poor nutrition knowledge. Biochemical parameters, including fasting blood sugar, total protein, lipid profile and white blood cell count, were in the normal ranges. Moreover, the majority of participants exhibited poor quality sleep that was accentuated among the participants undergoing rehabilitation, in addition to activity levels that were mainly low in the OST group. PWUD undergoing treatment for recovery in Lebanon are subject to various vulnerability factors creating challenges to treatment. Longitudinal assessments to better understand health problems arising during treatment and to identify the components of a comprehensive health promotion intervention during treatment for recovery are needed.

## Introduction

Illicit drug use is one of the most important public health hazards worldwide with 0⋅6 % of the world population suffering from severe drug use disorder. Specifically, among young people, illicit drug use has reached epidemic proportions^(1)^. Drug use corresponds with unhealthy lifestyle practices and often results in a variety of adverse social and health consequences^(2,3)^.

Once referred to treatment, whether via opioid substitution treatment (OST) or rehabilitation (detoxification or complete abstinence), addressing the lifestyle practices and improving the quality of life of people who use drugs (PWUD) seems to decrease the risk of relapse^(4)^. While undergoing treatment for recovery, a major shift occurs in the lifestyle of PWUD especially concerning nutrition and metabolism^(5)^.

The increasingly available time comes to be filled with overeating, often resulting in significant weight gain, varying at different recovery stages^(6–10)^. Yet, the intake of the majority of micronutrients remains below the recommended levels, which could be related to the increased intake of energy-dense foods, rather than nutrient-dense ones^(11,12)^. This issue remains understudied and the possibility of having hidden nutrient deficiencies in this population needs further investigations. The literature regarding the effect of treatment on metabolic parameters is also limited. After 6 months of methadone maintenance treatment (MMT), opioid addicts show metabolic disturbances, such as increased serum total cholesterol and low-density lipoproteins (LDL) compared with pre-treatment levels^(6,13)^. This elevation is associated with increased serum leptin levels and not with dietary intake and lifestyle practices.

The sleep of PWUD undergoing treatment, especially in rehabilitation services, has also received little attention in the scientific literature. The dearth of studies conducted on opiate addicts receiving MMT shows inadequate sleep quality and quantity, which could arise from a mix of causes, including psychopathological problems, nicotine use and duration of previous opiate use, in addition to methadone itself that produces sleep abnormalities^(14–16)^. During periods of drug withdrawal, total sleep time is decreased and sleep latency is increased. This disruption can persist for years post-treatment precipitating a possible relapse to addiction^(17,18)^. This issue is yet to be adequately addressed through large-scale studies across different treatment modalities.

Physical activity is another potential, non-pharmacological, element of treatment for addiction^(19)^: it reduces sufferings from withdrawals, anxiety and depression, in addition to improving self-confidence with a sense of the new quality of life^(4,20,21)^. Nevertheless, the controversy around the engagement of PWUD who are undergoing treatment for recovery in physical activity and the benefits of such activity remains in light of the scarcity of studies exploring this issue.

Treatment for substance use disorders mainly involves a combination of pharmacotherapy and psychotherapy approaches. Yet, there is a limited focus on improving nutrition and lifestyle practices that might enhance the outcomes of the treatment. While rehabilitation centres provide a controlled environment with the potential for offering healthy lifestyle practices due to the strict discipline in sleeping hours, mealtime, occupational tasks, and restricted access to television and social media, OST centres do not provide these comprehensive services and involve greater reliance on pharmacological tools for treatment^(22)^. Exploring the nutritional parameters and lifestyle practices of PWUD across both treatment modalities is essential to identify problematic areas and design targeted health promotion interventions.

Lebanon is a small high-middle income country in the Eastern Mediterranean region that suffered from internal and regional armed conflicts for more than three decades. These conflicts were predisposing factors for drug use due to its wide availability in the Lebanese market with the absence of control over its consumption^(1)^.

The present study aims to assess the nutritional parameters, namely nutritional status and anthropometrics, dietary intake, nutrition knowledge, food addiction and biochemical profiles, as well as different lifestyle practices, including sleep and physical activity, among PWUD undergoing treatment for recovery in Lebanon. We also focus on exploring the major significant differences in these parameters between the offered treatment modalities, namely OST and rehabilitation.

The in-patient residential rehabilitation centres are governed by the Ministry of Social Affairs in Lebanon. Acceptance is conditioned by a complete detoxification programme that is confirmed by a urine test prior to admission. The centres follow a strict discipline in terms of sleeping hours, meal times and tasks performed. Occasional supervised family visitation is allowed after 3 months of treatment initiation. Following each visit, a urine test is done to rule out the use of drugs. At the women's centre, children are not allowed to stay with their mothers and are only seen during visitation. The duration of treatment in the centres is 1 year.

The out-patient OST centres are under the jurisdiction of the Ministry of Public Health in Lebanon. Guidelines for acceptance are set by the ministry and include mainly previous failures in complete detoxification and rehabilitation. The patients visit the centre weekly to take the prescription of buprenorphine (opiate agonist) that is conditioned by a clean opiate urine test. Furthermore, random urine testing for buprenorphine is done to confirm the proper use of the medication. Treatment duration is individualised depending on the progress of the patient.

The team of care providers in both treatments consist mainly of social workers, psychologists and psychiatrists offering evidence-based behavioural therapy and pharmacotherapy. Medications are prescribed on an individual basis if needed and include: antidepressants, antipsychotics, bipolar drugs and others. Illicit drugs used by participants prior to treatment include: opiates, cannabis, stimulants, tranquillisers and barbiturates.

There is evidence of an increase in substance use in Lebanon from the onset of the civil war, particularly among the youth, with a prevalence higher than the global average^(23)^. Lebanon serves as a transit country for trafficking illicit drugs, in addition to local production and cultivation^(24)^. At the same time, Lebanon hosts WHO-designed knowledge hubs related to PWUD for the region^(24)^. The findings will inform the development of future targeted intervention programmes aimed at enhancing the lifestyle practices and improving the quality of life of PWUD undergoing treatment for recovery. They ultimately contribute to improving treatment outcomes and decreasing the risk of relapse.

## Methods

### Study design and population

We conducted this cross-sectional study in drug treatment facilities offering OST and institutionalised rehabilitation services post-detoxification in Lebanon. Randomly selecting the facilities was not an option since only three out of four OST centres and four out of seven rehabilitation centres operating in the country granted us entry permission. We, thus, targeted a convenience sample. We approached all PWUD receiving treatment in the OST and rehabilitation centres that granted us entry permission and informed them about the objectives, the methods of the study, and their right to withdraw at any time. The criteria for the participants to be included in the study were (1) to be Lebanese, (2) to be above 18 years of age and (3) to be receiving treatment for more than 1 month. In total, 369 people were approached, 214 were accepted to participate in the present study (response rate: 57⋅9 %) and 187 subjects met the inclusion criteria. The present study was conducted according to the guidelines laid down in the Declaration of Helsinki and all procedures involving research study participants were approved by the Lebanese International University's Committee on Research Ethics (CRE) (case number: LIUIRB-180122-NB). Written informed consent was obtained from all subjects.

### Sample size

We performed a statistical power analysis prior to the start of the study using the sample size and power analysis option of the Epi Info 7 software. Following an expected frequency of suboptimal nutritional status (the main outcome of interest) of 50 %, a 10 % confidence limit, a design effect of 1⋅5, and a confidence level of 95 %, 138 participants were needed (69 from OST and 69 from rehabilitation centres). Due to the lack of data on the frequency of malnutrition in this patient population, we used a frequency of 50 % to result in the largest sample size. We inflated the sample size by 20 % based on the response rate reported in similar studies (rehabilitation: 87–91 %^(25)^; OST: 80–90 %^(26)^), leading to a minimum required sample size of 166 participants. As 187 participants were included, the actual power was 92⋅6 %.

### Data collection

The present study took place between January 2018 and March 2019 in the treatment facilities. Trained licensed dietitians assessed the participants for anthropometrics, conducted the 24-h dietary recall and administered the questionnaires. All questionnaires were administered in Arabic (the native language of the participants). A licensed phlebotomist drew the blood samples, and a licensed nurse measured the blood pressure (BP). Data collection required 40–50 min per participant.

The study parameters included the following:

• Demographics, medical history and history of drug use were explored using a questionnaire focusing on socio-demographic characteristics, disease profile, medications, frequency and types of drugs used, duration of drug use and the type of drug treatment chosen. These questions were based on elements found in the literature associated with the nutritional status, eating habits and lifestyle of PWUD or those undergoing treatment for recovery.
• Nutritional status was assessed using the Subjective Global Assessment (SGA)^(27,28)^. The SGA is a clinical technique which assesses the nutritional status based on five features of the medical history (weight loss and its rate, dietary intake in relation to the participant's usual intake patterns, presence of significant gastro-intestinal symptoms, functional capacity and metabolic requirements of underlying disease) and four features of physical examination (loss of subcutaneous fat, muscle wasting, oedema and ascites). Based on the score of the above measurements, the nutritional status is classified as well-nourished (A), moderately malnourished (B) or severely malnourished (C).
• Self-reported weight change (kg) was assessed as the difference between reported usual pre-treatment body weight (kg) and measured body weight (kg) on the day of the assessment.
• Anthropometrics: (1) height (cm) using a portable digital wall mounted height scale measured to the nearest 0⋅1 cm without shoes; (2) weight (kg) using a calibrated mechanical floor scale without shoes and with light clothes on; (3) Body Mass Index (BMI) calculated as the ratio of weight (kg) and height squared (m^2^); (4) waist and neck circumferences measured to the nearest 0⋅1 cm, using a girth measuring tape; (5) body composition (%fat, %muscle mass and %visceral fat) measured with a BOCA X1 body composition analyzer (Medigate, Korea) and (6) BP (mmHg) using a standardised mercury sphygmomanometer (ALPK2, Japan) in the seated position after 5 min of rest, without prior smoking and exercise on that day. Two consecutive readings of systolic blood pressure (SBP) and diastolic blood pressure (DBP) were taken on the same arm within a 2-min interval. The mean of the two measurements was used for analysis.
• Dietary intake was assessed using the 24-h food recall using the United States Department of Agriculture's Multiple Pass Food Recall (MPR), which attenuates the recall bias^(29,30)^. In order to retrieve forgotten eating occasions and foods, the dietitian probed the participants more than once during the interview to provide comprehensive information about their intake and assist in portion size. Daily energy, macronutrient and micronutrient intake of the participants were computed from the 24-h recalls using the food composition database of the Nutritionist Pro software (Nutritionist Pro, Axxya Systems, San Bruno, CA, USA, version 5.1.0, 2018). The software from the database was expanded by adding an analysis of locally consumed foods and recipes^(31)^. Given that there are no gender or age-specific Dietary Reference Intakes (DRIs) for the Middle Eastern populations, values arising from the analysed data were compared with the US-based DRIs, as recommended by the Institute of Medicine (Dietary Reference Intake Tables).
• Nutrition knowledge was assessed using the Consumer-Oriented Nutrition Knowledge Questionnaire (CoNKQ) adapted from Spillmann and Keller^(32)^. This is a validated questionnaire, with good internal reliability (Cronbach's α is 0⋅743), criterion and construct validity. It consists of 20 comprehensive questions derived from consumer interviews and expert recommendations about healthy eating.
• Food addiction was assessed using the Yale Food Addiction Scale (YFAS)^(33)^. This is a highly reliable scale (Cronbach's α: 0⋅84) developed to identify individuals who are most likely to be exhibiting signs of addiction towards certain types of foods (high fat and high sugar). It consists of twenty-seven items that assess food patterns over the past 12 months and translates the criteria of substance dependence for at least 1 year in relation to eating behaviours including symptoms of tolerance and withdrawal, vulnerability in social activities, etc.
• Biochemical parameters: a blood sample of 5 ml was drawn and samples centrifuged directly by a portable tabletop machine and transported to the laboratory using a thermally insulated box. All blood collection was done early in the morning after an overnight fast after which breakfast was offered to the participants. Serum was analysed for complete blood count (CBC), fasting blood sugar (FBS, mg/dl), total protein (g/dl), serum albumin (g/dl), cholesterol (mg/dl), high-density lipoprotein-cholesterol (HDL, mg/dl), low-density lipoprotein-cholesterol (LDL, mg/dl), triacylglycerols (TAG, mg/dl), aspartate aminotransferase (AST, IU/L) and alanine aminotransferase (ALT, IU/L).
• Sleep quality was assessed using the Pittsburgh Sleep Quality Index (PSQI) developed by Buysse *et al.*^(34)^ This is a nine-item questionnaire, where four questions assess the duration of sleep, duration needed to fall asleep, the time needed to wake up and awake time spent in bed, in addition to five other questions assessing the reasons for troubled sleep. Answers are converted to a total score using an algorithm adapted from the developers of the questionnaire, with higher scores (≥5) indicating poor sleep quality and lower scores (0–4⋅9) indicating good sleep quality.
• Physical activity level was assessed using the International Physical Activity Questionnaire (IPAQ) short form^(35)^. The questionnaire consists of seven questions assessing the duration and frequency of light, moderate and vigorous physical activity completed in the past 7 d. The metabolic equivalent of tasks (METs) were calculated by multiplying the total minutes spent in the corresponding actions with the frequency (days) and the constants of 3⋅3, 4 and 8 for light, moderate and vigorous activity, respectively. The total MET value was computed by summing up the respective MET values for all activities that were done in bouts but were longer than 10 min in duration.

The Arabic version of the PSQI, culturally adapted by Haidar *et al.*^(36)^, was used; whereas the CoNKQ, YFAS and IPAQ were translated back and forth by two-independent expert bilingual translators. Furthermore, the translated versions of these questionnaires were pilot tested on a group of participants from different treatment centres for validation, the results of which were discarded^(37)^.

### Statistical analysis

We conducted a statistical analysis using the Statistical Package for Social Sciences (SPSS) version 21. We performed descriptive analyses to summarise the participants’ characteristics through means and standard deviation for continuous variables, and frequencies and percentages for categorical ones. Normality of the data was tested using the Kolmogorov–Smirnov test. We assessed the significant differences of study parameters between the two treatment modalities using *χ*^2^ test for categorical variables, independent samples *t* test for the continuous variables with the normal distribution, and Mann–Whitney *U* test for variables with the skewed distribution. We considered a *P*-value < 0⋅05 as statistically significant. The same analyses were conducted after excluding females participants, and differences in the results compared with the initial analyses (total sample) are reported.

## Results

### Demographics, medical history and history of drug use

In total, 187 PWUD undergoing treatment for recovery (OST: *n* 97; rehabilitation: *n* 90) participated in the study. Basic demographic information and medical history of the sample is presented in Table 1. Among the 187 participants, 92⋅0 % were males with the significant majority coming from the OST group (OST: 96⋅9 %; rehabilitation: 86⋅7 %; *P* < 0⋅05). The mean age of the participants was 32⋅0 ± 8⋅3 years, only 5⋅3 % were illiterate, and the majority received at least an intermediate level of education. One-quarter of the participants were using antidepressants (25⋅7 %); this finding was more common in the rehabilitation group (OST: 17⋅5 %; rehabilitation: 34⋅4 %; *P* < 0⋅05). This difference was no longer seen when females were excluded. More than one-third of them were on antipsychotic drugs (38⋅5 %) and 22⋅5 % on epilepsy-bipolar medications, with a significantly higher percentage of use in the rehabilitation group (OST: 11⋅3 %; rehabilitation: 34⋅4 %; *P* < 0⋅05).

**Table 1.** Demographic characteristics and medical history of the participants (*n* 187)

	OST (*n* 97)		Rehabilitation (*n* 90)		*P*-value	Total (*n* 187)	
	Mean	sd	Mean	sd		Mean	sd
Age (years)	33⋅7	8⋅2	30⋅2	8⋅1	0⋅002**	32⋅0	8⋅3
	*N*	%	*N*	%		*N*	%
Gender							
Male	94	96⋅9	78	86⋅7	0⋅010*	172	92⋅0
Female	3	3⋅1	12	13⋅3		15	8⋅0
Educational level							
Illiterate	8	8⋅2	2	2⋅2	0⋅267	10	5⋅3
Elementary/intermediate	35	36⋅1	31	34⋅4		66	35⋅3
Secondary	26	26⋅8	24	26⋅7		50	26⋅7
University	28	28⋅9	33	36⋅7		61	32⋅6
Occupation							
Unemployed/retired	40	41⋅2	49	54⋅4	0⋅019*^†^	89	47⋅6
Employed	28	28⋅9	16	17⋅8		44	23⋅5
Self-employed	29	29⋅9	20	22⋅2		49	26⋅2
Student	0	0⋅0	4	4⋅4		4	2⋅1
Other	0	0⋅0	1	1⋅1		1	0⋅5
Marital status							
Single	65	67⋅0	68	75⋅6	0⋅133	133	71⋅1
Married	24	24⋅7	12	13⋅3		36	19⋅3
Divorced/separated	8	8⋅2	10	11⋅1		18	9⋅6
Current housing							
Residence	97	100⋅0	25	27⋅8	<0⋅001*	122	65⋅2
Rehabilitation	0	0⋅0	65	72⋅2		65	34⋅8
People with whom the participant stays: pre-treatment (rehabilitation) and currently (OST)							
Alone	7	7⋅2	4	4⋅4	<0⋅001*	11	5⋅9
Spouse/partner	27	27⋅8	2	2⋅2		29	15⋅5
Parents	61	62⋅9	14	15⋅6		75	40⋅1
Relative/colleagues	2	2⋅1	66	73⋅3		68	36⋅4
No response	0	0⋅0	4	4⋅4		4	2⋅1
Medications used							
Antidepressents	17	17⋅5	31	34⋅4	0⋅008*^†^	48	25⋅7
Antipsychotic	31	32⋅0	41	45⋅6	0⋅056	72	38⋅5
Epilepsy-bipolar	11	11⋅3	31	34⋅4	<0⋅001*	42	22⋅5

OST, opioid substitution treatment.

* *P* < 0⋅05 using independent samples *t* test.

** *P* < 0⋅05 using Mann–Whitney *U* test.

† No significant difference between the OST and rehabilitation groups when females were excluded from the sample.

As evident in Table 2, as part of their history of drug use, 49⋅7 % of the participants used and injected drugs simultaneously and 79⋅7 % of them used drugs more than three times daily. There was a significant difference between the treatment modalities and both of these practices were more common among PWUD treated by OST (*P* < 0⋅05). Finally, 77⋅0 % of the participants were addicted only to drugs, while 16⋅0 % had alcohol drinking problems also, which was significantly more common in the rehabilitation group (OST: 1⋅0 %; rehabilitation: 32⋅2 %; *P* < 0⋅05).

**Table 2.** History of drug use of the particiants (*n* 187)

	OST (*n* 97)		Rehabilitation (*n* 90)		*P*-value	Total (*n* 187)	
	*N*	%	*N*	%		*N*	%
Type of drug previously used							
Drug use only	34	35⋅1	57	63⋅3	<0⋅001*	91	48⋅7
Drug injection only	1	1⋅0	1	1⋅1		2	1⋅1
Drug use and injection	62	63⋅9	31	34⋅4		93	49⋅7
No response	0	0⋅0	1	1⋅1		1	0⋅5
Frequency of drug previously used or injected							
Up to three times daily	85	87⋅6	64	71⋅1	0⋅034*	149	79⋅7
Once or more daily	9	9⋅3	17	18⋅9		26	13⋅9
Once or more weekly	3	3⋅1	6	6⋅7		9	4⋅8
Does not know or remember	0	0⋅0	1	1⋅1		1	0⋅5
No response	0	0⋅0	2	2⋅2		2	1⋅1
Previous treatment							
None	37	38⋅1	49	54⋅4	<0⋅001*	86	46⋅0
OST	6	6⋅2	6	6⋅7		12	6⋅4
Rehabilitation	22	22⋅7	28	31⋅1		50	26⋅7
Rehabilitation and OST	10	10⋅3	4	4⋅4		14	7⋅5
Hospital detoxification	18	18⋅6	2	2⋅2		20	10⋅7
Hospital detoxification and rehabilitation	4	4⋅1	0	0⋅0		4	2⋅1
No response	0	0⋅0	1	1⋅1		1	0⋅5
Other addiction							
None	90	92⋅8	54	60⋅0	<0⋅001*	144	77⋅0
Alcohol	1	1⋅0	29	32⋅2		30	16⋅0
Other	6	6⋅2	7	7⋅8		13	7⋅0
	Mean	sd	Mean	sd		Mean	sd
Duration of drug use (years)	11⋅4	7⋅2	10⋅9	7⋅3	0⋅632	11⋅2	7⋅2
Duration of drug injection (years) (among those who reported drug injection)	7⋅3	6⋅3	8⋅0	5⋅6	0⋅469	7⋅5	6⋅1
Age at first drug use and/or injection (years)	18⋅3	6⋅7	16⋅3	4⋅6	0⋅006**	17⋅4	5⋅9
Number of previous treatment attempts	3⋅6	4⋅9	2⋅0	2⋅3	0⋅580	3⋅1	4⋅3
Treatment duration (months)	31⋅0	25⋅0	5⋅7	5⋅3	0⋅000**	19⋅2	23⋅0

OST, opioid substitution treatment.

* *P* < 0⋅05 using independent samples *t* test.

** *P* < 0⋅05 using Mann–Whitney *U* test.

### Nutritional status, weight gain and anthropometric measurements

The vast majority of the participants (88⋅8 %) were well nourished based on the SGA, and only 11⋅2 % of them fell in the moderately malnourished category.

Two-thirds (66⋅8 %) of the participants reported weight gain during treatment. This finding was more common in the rehabilitation group (OST: 54⋅6 %; rehabilitation: 80⋅0 %; *P* < 0⋅05). On the other hand, 20⋅0 % of the sample reported weight loss. This was more common in the OST group (OST: 32⋅0 %; rehabilitation: 8⋅9 %; *P* < 0⋅05). On average, the BMI of the participants increased from 24⋅9 ± 4⋅8 kg/m^2^ pre-treatment to 27⋅0 ± 4⋅9 kg/m^2^ during treatment. Other anthropometric measurements are detailed in Table 3.

**Table 3.** Nutritional status, weight change and anthropometric measurements of the participants (*n* 187)

	OST (*n* 97)		Rehabilitation (*n* 90)		*P*-value	Total (*n* 187)	
	*N*	%	*N*	%		*N*	%
Subjective Global Assessment (SGA)							
Well nourished	86	88⋅7	80	88⋅9	0⋅960	166	88⋅8
Moderately malnourished	11	11⋅3	10	11⋅1		21	11⋅2
Weight change							
Weight loss	31	32⋅0	8	8⋅9	<0⋅001*	39	20⋅9
No change	13	13⋅4	10	11⋅1		23	12⋅3
Weight gain	53	54⋅6	72	80⋅0		125	66⋅8
	Mean	sd	Mean	sd		Mean	sd
Pre-treatment BMI (kg/m^2^)	25⋅9	4⋅6	23⋅0	4⋅9	0⋅002**	24⋅9	4⋅8
During treatment BMI (kg/m^2^)	26⋅6	5⋅2	27⋅5	4⋅5	0⋅189	27⋅0	4⋅9
SBP (mmHg)	123⋅7	13⋅0	126⋅5	15⋅3	0⋅228	125⋅0	14⋅2
DBP (mmHg)	75⋅7	11⋅3	79⋅0	15⋅9	0⋅110^†^	77⋅3	13⋅7
Percent body fat (%)	25⋅0	8⋅4	26⋅2	7⋅3	0⋅313	25⋅6	7⋅9
Waist circumference (cm)	91⋅0	14⋅1	93⋅4	12⋅2	0⋅218	92⋅2	13⋅2
Neck circumference (cm)	37⋅4	3⋅2	37⋅6	3⋅4	0⋅770	37⋅5	3⋅3
Male participants							
Percent body fat (%)	24⋅8	8⋅4	24⋅9	6⋅7	0⋅935	25⋅9	7⋅7
Waist circumference (cm)	91⋅3	14⋅0	94⋅6	12⋅5	0⋅024**	92⋅8	13⋅4
Female participants							
Percent body fat (%)	31⋅4	6⋅5	34⋅6	4⋅9	0⋅356	33⋅9	5⋅2
Waist circumference (cm)	81⋅6	15⋅3	85⋅5	6⋅1	0⋅705	84⋅7	8⋅1

OST, opioid substitution treatment; BMI, body mass index; SBP, systolic blood pressure; DBP, diastolic blood pressure.

* *P* < 0⋅05 using independent samples *t* test.

** *P* < 0⋅05 using Mann–Whitney *U* test.

† Significant difference between the OST and rehabilitation groups when females were excluded from the sample.

### Dietary intake

The energy, macro- and micronutrient intakes reported by the participants are detailed in Table 4. The mean daily energy intake was 32⋅8 ± 18⋅3 calories (kcal) per kg of body weight, and 62⋅3 % of our sample reported consuming more than 25 kcal per kg of body weight. The average daily intake of proteins was 1⋅0 ± 0⋅6 g per kg of body weight with higher intakes reported by the OST group (OST: 1⋅1 ± 0⋅7 g/kg; rehabilitation: 0⋅9 ± 0⋅5 g/kg; *P* < 0⋅05). Furthermore, 41⋅5 % of the participants had intakes below 0⋅8 g of proteins per kg body weight; this finding was more noted in the rehabilitation group (OST: 34⋅4 %; rehabilitation: 48⋅9 %; *P* < 0⋅05).

**Table 4.** Intake of energy, macro- and micronutrients of the participants (*n* 187)

	OST (*n* 97)		Rehabilitation (*n* 90)		*P*-value	Total (*n* 187)	
	Mean	sd	Mean	sd		Mean	sd
Energy (kcal)	2728⋅6	1494⋅7	2471⋅2	1013⋅5	0⋅173^‡^	2602⋅0	1283⋅8
Energy (kcal/kg)	34⋅8	21⋅1	30⋅8	14⋅7	0⋅140	32⋅8	18⋅3
Protein (g)	91⋅9	52⋅4	73⋅2	39⋅6	0⋅005*	82⋅7	47⋅4
Protein (g/kg)	1⋅1	0⋅7	0⋅9	0⋅5	0⋅006*	1⋅0	0⋅6
Added sugar (g)	95⋅9	94⋅7	87⋅2	76⋅1	0⋅091	91⋅6	85⋅9
Fibre (g)	21⋅8	13⋅4	21⋅2	10⋅3	0⋅657	21⋅5	11⋅9
Calcium (mg)	816⋅2	571⋅4	736⋅6	425⋅2	0⋅815	777⋅1	505⋅0
Potassium (mg)	2761⋅9	1584⋅7	2300⋅2	1150⋅1	0⋅025*^†^	2534⋅8	1403⋅5
Iron (mg)	19⋅4	39⋅0	14⋅7	8⋅1	0⋅569	17⋅1	28⋅4
Zinc (mg)	11⋅9	9⋅2	10⋅2	6⋅1	0⋅133	11⋅1	7⋅9
Magnesium (mg)	322⋅0	242⋅7	278⋅3	116⋅2	0⋅916	300⋅5	192⋅0
Selenium (mcg)	118⋅3	75⋅6	94⋅3	54⋅1	0⋅028**	106⋅5	66⋅8
Thiamin (mg)	1⋅9	1⋅1	1⋅7	0⋅8	0⋅125	1⋅8	0⋅9
Riboflavin (mg)	1⋅8	1⋅3	1⋅5	0⋅7	0⋅197	1⋅7	1⋅1
Niacin (mg)	25⋅7	19⋅6	19⋅8	14⋅9	0⋅027**	22⋅8	17⋅7
Vitamin C (mg)	93⋅7	98⋅0	85⋅1	81⋅0	0⋅944	89⋅5	89⋅9
Vitamin A (mcg)	826⋅1	1724⋅7	431⋅4	360⋅0	0⋅336	632⋅0	1267⋅3
Vitamin D (mcg)	1⋅7	4⋅6	1⋅2	1⋅6	0⋅406	1⋅5	3⋅5
Vitamin E (mg)	10⋅8	9⋅6	9⋅9	5⋅4	0⋅619	10⋅3	7⋅8
Pyridoxine (mg)	1⋅6	1⋅1	1⋅2	0⋅7	0⋅001**	1⋅4	0⋅9
Folate (mcg)	355⋅3	338⋅3	316⋅3	240⋅0	0⋅731	336⋅1	293⋅9
	*N*	%	*N*	%		*N*	%
Categories of energy intake							
<25 kcal/kg	36	38⋅7	33	36	0⋅776	69	37⋅7
≥25 kcal/kg	57	61⋅3	57	63		114	62⋅3
Categories of protein intake							
<0⋅8 g/kg	32	34⋅4	44	48⋅9	0⋅047*	76	41⋅5
≥0⋅8 g/kg	61	65⋅6	46	51⋅1		107	58⋅5

OST, opioid substitution treatment.

* *P* < 0⋅05 using independent samples *t* test.

** *P* < 0⋅05 using Mann–Whitney *U* test.

† No significant difference between the OST and rehabilitation groups when females were excluded from the sample.

‡ Significant difference between the OST and rehabilitation groups when females were excluded from the sample.

Looking at micronutrients, potassium was the only micronutrient showing a significant difference between the two groups (OST: 2761⋅9 ± 1584⋅7 mg; rehabilitation: 2300⋅2 ± 1150⋅1 mg; *P* < 0⋅05). This difference was no longer seen when females were excluded.

### Biochemical parameters

The biochemical profile of the participants is detailed in Table 5. The mean values of HDL and LDL were 43⋅8 ± 12⋅3 and 115⋅5 ± 38⋅9 mg/dl, respectively, with no statistical difference between the treatment groups (*P* > 0⋅05). The mean value of FBS and total serum proteins were 89⋅7 ± 13⋅6 mg/dl and 7⋅3 ± 0⋅4 g/dl, respectively. Both results were significantly higher in the OST group (*P* < 0⋅05).

**Table 5.** Biochemical parameters of the participants (*n* 187)

	OST (*n* 97)		Rehabilitation (*n* 90)		*P*-value	Total (*n* 187)	
	Mean	sd	Mean	sd		Mean	sd
Erythrocyte (cells/mcl)	5⋅2	0⋅5	5⋅2	0⋅4	0⋅229	5⋅2	0⋅4
Hgb (g/dl)	14⋅7	1⋅5	15⋅0	1⋅2	0⋅127^‡^	14⋅8	1⋅3
Hct (%)	43⋅6	3⋅7	44⋅5	2⋅9	0⋅085*	44⋅0	3⋅4
Leucocyte (cells/mcl)	8⋅5	2⋅5	7⋅2	2⋅1	0⋅001*	7⋅9	2⋅4
Platelet (cells/mcl)	259⋅8	63⋅5	249⋅2	66⋅2	0⋅291	254⋅4	64⋅9
Total Proteins (g/dl)	7⋅4	0⋅4	7⋅3	0⋅4	0⋅008*^†^	7⋅3	0⋅4
Albumin (g/dl)	4⋅3	0⋅2	4⋅2	0⋅3	0⋅135	4⋅3	0⋅3
FBS (mg/dl)	94⋅2	16⋅8	85⋅4	7⋅4	<0⋅001*	89⋅7	13⋅6
Total cholesterol (mg/dl)	188⋅8	53⋅4	190⋅9	39⋅8	0⋅773	189⋅9	46⋅9
LDL (mg/dl)	115⋅1	44⋅3	115⋅9	33⋅1	0⋅891	115⋅5	38⋅9
HDL (mg/dl)	43⋅3	12⋅3	44⋅3	12⋅5	0⋅339	43⋅8	12⋅3
Males	43⋅0	11⋅8	43⋅8	12⋅8	0⋅554	43⋅4	12⋅3
Females	51⋅0	23⋅8	47⋅5	9⋅7	0⋅829	48⋅2	12⋅5
TAG (mg/dl)	118⋅0	69⋅2	129⋅3	147⋅4	0⋅527	123⋅8	115⋅6
ALT (IU/l)	37⋅6	50⋅7	32⋅3	26⋅5	0⋅870	34⋅9	40⋅2
AST (IU/l)	32⋅7	29⋅7	32⋅1	25⋅2	0⋅924	32⋅4	27⋅4

OST, opioid substitution treatment; Hgb, haemoglobin; Hct, haematocrit; FBS, fasting blood sugar; LDL, low-density lipoprotein; HDL, high-density lipoprotein; TAG, triacylglycerol; ALT, alanine aminotransferase; AST, aspartate aminotransferase.

* *P* < 0⋅05 using independent samples *t* test.

**P < 0⋅05 using Mann–Whitney *U* test.

† No significant difference between the OST and rehabilitation groups when females were excluded from the sample.

‡ Significant difference between the OST and rehabilitation groups when females were excluded from the sample.

### Lifestyle practices

Table 6 presents the lifestyle practices of the participants. More than three-quarters (75⋅3 %) of our sample had a poor quality of sleep; this was a more common finding in the rehabilitation group (OST: 68⋅8 %; rehabilitation: 82⋅2 %; *P* < 0⋅05). This difference was no longer seen when females were excluded. Furthermore, half of the participants (49⋅2 %) had a low physical activity level that was significantly higher in the OST group (OST: 71⋅1 %; rehabilitation: 25⋅6 %; *P* < 0⋅05). Interestingly, approximately half of the participants (48⋅9 %) were diagnosed with food addiction and more than two-thirds of our sample (69⋅5 %) showed poor knowledge of nutrition; however, no significant differences were found between the two groups regarding both parameters.

**Table 6.** Lifestyle practices: sleep, physical activity levels, food dependence and nutrition knowledge of the participants (*n* 187)

	OST (*n* 97)		Rehabilitation (*n* 90)		*P*-value	Total (*n* 187)	
	*N*	%	*N*	%		*N*	%
Sleep quality index							
Good sleep quality	30	31⋅3	16	17	0⋅033*^†^	46	24⋅7
Poor sleep quality	66	68⋅8	74	82		140	75⋅3
Physical activity level							
Low activity level	69	71⋅1	23	25⋅6	<0⋅001*	92	49⋅2
Moderate activity level	20	20⋅6	28	31⋅1		48	25⋅7
High activity level	8	8⋅2	39	43⋅3		47	25⋅1
Food addiction							
No diagnosis met	53	54⋅6	40	47⋅1	0⋅307	93	51⋅1
Diagnosis met	44	45⋅4	45	52⋅9		89	48⋅9
Nutrition knowledge							
Poor nutrition knowledge	71	73⋅2	59	65⋅6	0⋅257	130	69⋅5
Good nutrition knowledge	26	26⋅8	31	34⋅4		57	30⋅5

OST, opioid substitution treatment.

* *P* < 0⋅05 using independent samples *t* test.

**P < 0⋅05 using Mann–Whitney *U* test.

† No significant difference between the OST and rehabilitation groups when females were excluded from the sample.

## Discussion

To date, the nutritional parameters and lifestyle practices of PWUD undergoing treatment for recovery have received little attention in the scientific literature. Up to our knowledge, the present study was the first to assess these variables among PWUD undergoing treatment for recovery in Lebanon and the Middle East region. Examining these parameters in the region is important since PWUD, undergoing treatment in rehabilitation centres in Lebanon, reported excessive food intake as a diversion from the frustration imposed by the strict environment and lack of leisure activities in treatment centres. Furthermore, physical activity was a mandatory routine that was not enjoyed^(38)^. Besides, reports on drug use in the region do not tackle any of the lifestyle or nutritional parameters covered in the present study^(23,24)^. Accordingly, the present study pioneered in exploring major significant differences in the nutritional parameters and lifestyle practices across treatment modalities, namely OST and rehabilitation. Our sample mainly consisted of male participants with early drug use initiation and high frequency of pre-treatment drug use. They are currently exhibiting polymedication as part of their treatment. Our results showed that PWUD undergoing treatment for recovery are subject to numerous vulnerability factors, namely excessive weight gain, poor nutrition knowledge, high food addiction level, in addition to poor sleep quality, and low physical activity level.

First, our findings pinpoint overnutrition in this population group. The vast majority of the participants in both treatment modalities showed good nutritional status, as assessed by the SGA. Furthermore, about 67 % of this population, specifically those undergoing rehabilitation, gained weight during treatment. Most importantly, the mean BMI of the group increased to reach the overweight category, and the adiposity in both genders was above the recommended range. Our results provide further evidence regarding the weight gain and increase in BMI seen among PWUD in the months following entry into treatment. This was specifically among PWUD undergoing MMT^(6,7,10,39,40)^ and residential rehabilitation programme^(41–43)^. The increase in weight, which was more common in the rehabilitation group, could be also attributed to the structured meals offered in the in-patient residential centres, the cravings for sweets as a replacement for drugs, and the excessive eating as a diversion from the frustration of the strict environment imposed^(38,44)^. Furthermore, this weight gain was perceived as a sign of health to compensate for the weight lost during addiction^(38)^. Excessive weight gain promotes risk for a variety of health outcomes and remains of a potential clinical and medical significance. Additionally, body dissatisfaction, usually arising from overweight, may be a trigger relapse, especially among females who use drugs^(10,44,45)^. This increase in weight, high adiposity and overnutrition comes in parallel with the high reported energy intake among the participants undergoing both treatment modalities. The short duration of treatment among our participants, mainly in the rehabilitation group, may explain this weight gain observed. Available studies confirm increased weight gain and binge eating during the early phase of treatment, as opposed to a more structured pattern in the latter phase, which typically occurs 6 months post-treatment entry^(41,44)^. Regardless of the precise mechanism involved in the weight gain among this population group, identifying predictors of weight gain would be extremely helpful allowing for preventive measures to be adopted during treatment.

Interestingly, the mean BMI for the participants increased from the normal category prior to treatment (18⋅0–24⋅9 kg/m^2^) to the overweight category during treatment (25⋅0–29⋅9 kg/m^2^). This is coherent with the findings of Fenn *et al.*^(46)^, but stands in contrast to prior suggestions that weight increase during treatment may be due to a malnourished state moving towards a healthier weight^(47,48)^. Documented longitudinal assessment of weight gain and adiposity pre- and during treatment is needed to better understand this issue.

To assess the participants’ nutritional status, anthropometric indices alone are not the best indicator. This population group displays hidden deficiencies and disturbed metabolic parameters that need to be deeply investigated by biochemical data, nutrition focused-physical findings, in addition to food addiction^(44,49)^. Furthermore, validated tools to assess the nutritional status of this population group need to be developed.

Besides, the present study revealed poor nutrition knowledge and a high rate of food addiction among the majority of our participants. These two parameters have not been largely studied among PWUD undergoing treatment for recovery. Similarly, subjects undergoing MMT who scored low on knowledge about healthy diet showed a higher preference for energy-dense foods and had a higher BMI^(39)^. In contrast to our results, Sason *et al.*^(8)^ reported good knowledge of basic nutrition by all of the participants undergoing MMT. Furthermore, 10 % of their patients were diagnosed with food addiction. A reason behind the latter finding could be attributed to the exclusion of participants with a BMI < 26 kg/m^2^ and those with good nutrition knowledge in that study. Emerging research shows conflicting results correlating food addiction and weight gain among overweight and obese individuals in the general population. Some studies show a positive relation, while others show none^(50–53)^. It has been suggested that an addiction to food could act in a similar way to other substance addictions. Repeated exposures to pleasurable food would diminish the dopamine brain response^(54,55)^. This would lead to larger quantities of food consumed in order to feel satisfied, subsequently perpetuating overeating^(56)^. Based on this, the high rate of participants diagnosed with food addiction in our study could potentially explain the weight gain reported.

The weight gain seen among patients undergoing treatment from drug use may not be solely due to changes in eating behaviours. Pharmacological treatments received by the participants or the maintenance treatment with the partial opioid agonist may have a significant effect. Weight gain is a frequently observed side effect with many antipsychotic treatments and seems to be underreported and underrecognized in many patients^(57,58)^. Interestingly, our study supports this finding, where the highest percentage of weight gain seen in the rehabilitation centres could be attributed to the high intake of antipsychotic drugs taken by the participants.

Notably, there was a significant difference in weight gain pattern between the two treatment modalities. Rehabilitation participants showed a much greater weight gain and increase in BMI that was present in about 80 % of the subjects. Mysels *et al.*^(59)^ did not detect a statistical significant course of weight gain between methadone and naltrexone maintenance treatment.

Despite the fact that the majority of the studies in this population group focusses on weight gain, there was a considerable variety of weight changes seen among our participants. Approximately one-third of the participants experienced either weight loss (21 %) or no weight change (12 %). This finding was more apparent among the OST participants and could be attributed to the limited economic resources and the financial burdens of family support^(60)^. The patterns, determinants, outcomes of nutritional status, weight gain or loss in the population of PWUD undergoing treatment for recovery, and the differences noted between OST and rehabilitation need to be explored by future studies. This will inform the development of targeted prevention and intervention programmes to improve PWUD's nutritional health and wellbeing along the recovery process.

Regarding macro- and micronutrient intake of our population, the majority of the participants had high energy and protein intakes potentially justifying the increase in weight reported. This is consistent with other studies that showed an increase in the overall intake of energy and proteins after initiation of the treatment^(12,61)^. Interestingly, the majority of the vitamins and minerals were within the recommended levels of intake. This can be explained by the structured healthy meals offered in the rehabilitation centres as described by some of the participants in our qualitative research with this population group^(38)^. On the other hand, our results come in contrast to other studies indicating low levels of micronutrients in different treatment modalities. Their finding was mainly attributed to the increased intakes of energy-dense foods, rather than nutrient-dense ones^(11,12,59)^. Nevertheless, assessing the nutritional status of PWUD only by anthropometric measurements and dietary intake may not reveal severe malnutrition, as hidden deficiencies might exist and can only be detected through measuring plasma nutrients^(2,44)^.

To date, few studies have investigated selected biochemical indices in this patient population, and up to our knowledge, no studies on biochemical indices were done specifically on PWUD in rehabilitation centres. Our participants had, on average, normal values of lipid profile during treatment which is coherent with the available studies in PWUD undergoing OST^(6,13)^. Additionally, FBS, total proteins and leucocytes were in the normal category. In contrast to other findings in PWUD undergoing detoxification, our participants showed normal levels of haemoglobin and albumin^(62)^. This could be attributed to the high micronutrient intakes observed by our participants as opposed to the low intake of nutrient-dense foods reported elsewhere^(63)^. Furthermore, while FBS was within the normal range, it is important to note that it was significantly higher in the OST group. This finding can be supported by the fact that chronic administration of opiate agonists can cause insulin resistance, abnormalities in glucose metabolism, in addition to a higher risk of developing diabetes^(64,65)^. This again highlights the need for further comparative studies between the two treatment modalities. Additionally, longitudinal studies examining the changes of these biochemical indices throughout the treatment and their implications on disease development are needed.

Looking at lifestyle practices, our population exhibited an overall poor quality of sleep. This was more accentuated among the participants undergoing rehabilitation. The sleep of PWUD undergoing treatment for recovery, especially in rehabilitation services, was rarely investigated by the scientific literature. It can be hypothesised that the poor quality of sleep observed among the participants in rehabilitation could be attributed to the drug withdrawal symptoms^(18)^. Most of the literature confirms poor sleep quality among patients in MMT^(15,16,66)^. Sleep disturbances could be attributed to the comorbid conditions present among this population like psychopathology, alcohol and nicotine abuse, in addition to methadone itself^(14,16)^. Generally, sleep is an important aspect of the health-related quality of life of people undergoing treatment for drug use. It may lead to poor treatment adherence, opiate relapse and the risk of sedative abuse^(14,15,67)^. Furthermore, studies confirm a positive association between short sleep duration and weight gain among healthy adults^(68,69)^. Relating poor quality of sleep to weight gain reported and investigating its outcomes among this population group should be further investigated.

Finally, half of the participants in our sample, mainly in the OST group, had a low physical activity level. This significant difference between the two groups could be attributed to the mandatory daily exercise offered in rehabilitation centres as part of the daily routines. Data from our qualitative work on this population group in rehabilitation centres showed contradictory perceptions regarding the mandatory physical activity programmes offered^(38)^. Some reported that it improved their physical and psychological wellbeing and decreased their drug cravings, while others described it as a boring routine that needs to be personalised. Physical activity was shown to reduce sufferings from withdrawals, anxiety and depression, in addition to improving self-confidence^(4,20)^. Furthermore, Brown *et al.*^(70)^ showed that physical activity, as an adjunct to the treatment in this population group, resulted in increasing days of abstinence from drug and alcohol use. However, recommendations on the type and duration of the physical activity to promote these benefits need further exploration due to the paucity of studies in the literature. Additionally, the possible correlation between low physical activity level and weight gain seen in this group needs to be further studied.

### Strengths and limitations

The present study presents numerous strengths. First, it pioneers in providing insights into the nutritional parameters and lifestyle practices among a vulnerable population, namely PWUD undergoing treatment for recovery. This was the first study to explore these issues in participants from the Middle East region, specifically Lebanon, where several factors promote drug use including the decades of internal and regional armed conflicts^(71,72)^. Second, the present study highlights a gap in the literature regarding the need for comparison studies between the different treatment modalities offered. Third, we used an exhaustive sample, where all treatment centres were approached. And, in those who granted us permission of entry, all individuals receiving the treatment were approached for participation. Fourth, data collection was conducted by licensed dietitians, nurses and phlebotomists using calibrated instruments. Furthermore, biochemical parameters were analysed in a laboratory certified by the Ministry of Public health in Lebanon. Additionally, we used validated assessment tools such as SGA^(27,28)^, CoNKQ^(32)^, YFAS^(33)^, PSQI^(34)^ and IPAQ^(35)^, although further validation among PWUD is required. Finally, the present paper paves the way to further studies assessing the nutritional status and intake to identify the predictors of weight gain of PWUD undergoing treatment for recovery. Additionally, it highlights the need for an in-depth assessment of lifestyle factors among this population group, resulting in a more comprehensive intervention within treatment centres to promote recovery.

In contrast, the present study had some limitations. First, female participants were less represented in our sample. A major reason for this, as mentioned earlier, was the limited number of residential centres for females in Lebanon, and the fear of stigma among females receiving OST, which prevented them from participating in the present study. Second, the reported pre-treatment weight and BMI were used, and subsequently, weight change was reported. As this was a cross-sectional study, participants were only met at the date of data collection. Also, change in usual dietary intake (a component of the SGA) was reported by the participant, and it was not assessed objectively. Similarly, dietary intake was measured once using 24-h dietary recall which does not estimate the usual food intake and depends on the memory of the participants. We tried to attenuate the recall bias by using the United States Department of Agriculture's MPR^(29,30)^. In addition, physical activity and sleep quality were reported, but not measured. Third, given the cross-sectional nature of the present study, no baseline data for biochemical and nutritional indices were present to compare our results and evaluate the effect of the treatment on disease development and risk of relapse. Fourth, it is possible that because participants were still actively involved in the rehabilitation services when collecting the data, their responses may have been more socially desirable to avoid offending their host institution. Fifth, there is a limited generalizability of the results, because of the non-random sample due to the restrictions by the centres discussed earlier. Sixth, the Arabic versions of the translated questionnaires used in the study need to be validated for future use, although translated back and forth by two-independent bilingual translators. Furthermore, the present study lacks a comparison against an aged-matched control group due to the lack of data regarding the variables of interest in the general Lebanese population. Finally, our results should be confirmed by future studies in other countries. This will inform whether our findings are shared features among this patient population or unique characteristics of PWUD treated in Lebanon.

### Future studies

Longitudinal studies are needed to further examine changes in biochemical indices, lifestyle practices, adiposity and weight throughout the recovery process and across treatment modalities, as well as the implications of these issues on treatment outcomes and disease development. Specifically, the patterns, determinants and outcomes predictors of weight change need to be investigated to inform preventive measures during treatment. Furthermore, the type and duration of the physical activity to be adopted throughout recovery need further exploration. Finally, although the results obtained by the present study provide insights for health promotion intervention to be implemented in drug treatment centres, focusing on the importance of increasing nutrition knowledge and physical activity, improving sleep and dealing with food addiction need to be further studied. Its effectiveness and cost-effectiveness should also be assessed.

## Conclusion

The present study fills a gap in the literature regarding the nutritional parameters and lifestyle practices among PWUD in different treatment modalities. The results obtained provide evidence that PWUD undergoing treatment for recovery have a good nutritional status, but experience suboptimal dietary intake, weight gain and increased adiposity. They also have poor lifestyle practices, specifically poor quality of sleep and low physical activity levels. Further research should be conducted on a more representative sample to examine the correlation between specific nutritional parameters and lifestyle practices with weight gain, disease development and risk of relapse across different treatment modalities. Additional research is also needed to identify the components of a comprehensive and targeted health promotion intervention to be implemented during treatment to improve PWUD's nutritional health and wellbeing throughout the recovery process. This vulnerable group faces many challenges in maintaining a healthy lifestyle, and health promotion programmes are essential to improve the treatment experience and prevent relapse.
